# Diagnostic performance of gait speed, G8 and G8 modified indices to screen for vulnerability in older cancer patients: the prospective PF-EC cohort study

**DOI:** 10.18632/oncotarget.17361

**Published:** 2017-04-21

**Authors:** Frederic Pamoukdjian, Florence Canoui-Poitrine, Coralie Longelin-Lombard, Thomas Aparicio, Nathalie Ganne, Philippe Wind, Claudia Martinez-Tapia, Etienne Audureau, Georges Sebbane, Laurent Zelek, Elena Paillaud

**Affiliations:** ^1^ Geriatric Department, APHP, Avicenne Hospital, Coordination Unit in Geriatric Oncology, Bobigny, France; ^2^ Université Paris 13, Sorbonne Paris Cité, Laboratoire Educations et Pratiques de Santé, Bobigny, France; ^3^ Public Health Department, APHP, Henri-Mondor Hospital, Créteil, France; ^4^ Université Paris-Est, UPEC, DHU A-TVB, IMRB- EA 7376 CEpiA, Clinical Epidemiology And Ageing Unit, Créteil, France; ^5^ Gastroenterology Department, APHP, Avicenne Hospital, Bobigny, France; ^6^ Hepatology Department, APHP, Jean Verdier Hospital, Bondy, France; ^7^ Université Paris 13, Sorbonne Paris Cité, “Equipe Labellisée Ligue Contre le Cancer”, Bobigny, France; ^8^ Inserm, UMR-1162, "Functional Genomics of Solid Tumors", Paris, France; ^9^ APHP, Avicenne Hospital, Surgery Department, Bobigny, France; ^10^ Department of Medical Oncology, APHP, Avicenne Hospital, Bobigny, France; ^11^ Geriatric Department, APHP, Henri-Mondor Hospital, Geriatric Oncology Unit, Créteil, France

**Keywords:** geriatric assessment, cancer, vulnerability, gait speed, G8 index, Gerotarget

## Abstract

**Background:**

The diagnostic performance of tools used to screen vulnerability in older cancer patients varies widely. We assessed the diagnostic performance of gait speed (GS) for assessing vulnerability in such patients.

**Methods:**

All consecutive outpatients 65 years and older were referred for geriatric oncology assessment (GA) before a therapeutic decision between November 2013 and April 2016 in a bicentric observational and prospective cohort study. Vulnerability was defined as impaired score on at least one of the 6 domains of the GA. GS and the G8 index and G8 modified index were assessed at the first geriatric oncology visit during the GA. Sensitivity, specificity, positive and negative predictive value and positive and negative likelihood ratio were estimated. The accuracy of the three tools was analysed by the area under the receiver operating characteristic curve (AUC).

**Results:**

Among 269 included patients (mean [SD] age, 81.3 years [5.9]; 55% women, 94.4% solid tumors; 39.4% with metastasis), 252 (93.7%) had impaired GA. With the GS threshold of 1 m/s, sensitivity was 79.4% (95% CI, 73.8-84.2), specificity 64.7% (38.3-85.8), and AUC 82.0 (74.0-90.0). The corresponding values for the G8 index were 90.1% (85.7-93.5), 35.3% (14.2-61.7), and 79.0 (70.0-88.0) and G8 modified index were 89.3% (84.8-92.8), 64.7% (38.3-85.8), and 84.0 (74.0-92.0).

**Conclusions:**

GS < 1 m/s with a single measure could be used as a new screening tool for detecting vulnerability in older cancer outpatients. This first external validation of the G8 modified index was very good.

## INTRODUCTION

With the ageing of society, oncologists will see increasing numbers of older cancer patients. Indeed, older cancer patients present 60% to 70% of newly diagnosed cancers. Treatment guidelines are based on clinical studies, from which older patients have often been excluded [1], so the transportability of the guidelines to older patients is uncertain.

This older cancer population is heterogeneous in comorbidities, physical reserve, functional status and socioeconomic environment [2]. Geriatric assessment (GA) is recommended by the International Society of Geriatric Oncology (SIOG) [3] and has been used to detect disabilities and comorbid conditions that could contribute to an older patient's vulnerability, predisposing to poor outcomes and treatment complications [4].

However, GA is time- and resource-consuming and not necessary for all patients, so physicians have developed shorter screening tools to identify patients who need a GA in a multidisciplinary approach. These screening tools could distinguish fit older cancer patients, who are able to receive standard cancer treatment, from vulnerable patients, who should receive a full assessment to determine the most appropriate treatment plan [3].

Several screening tools for vulnerability have been developed, but their performance varies widely. Recently, the performance of 17 different screening tools was assessed, in 22 studies involving a total of 5950 older cancer patients (median age 65-79 years, with various cancer types). Vulnerability was defined by at least one or two impaired GA domains. Among these tools, the G8 index was the most promising, with sensitivity greater than 80%, specificity greater than 60% and negative predictive value (NPV) 35% to 78% [3]. More recently, the G8 index was optimized with only six items. This G8 modified index was validated with 414 older cancer patients (median age 81 years, with various solid and haematological cancers) with better sensitivity (89.2%) and specificity (79%) than the G8 index and NPV 52.8% [5].

In a systematic review [6], we suggested the use of gait speed (GS) as screening tool for vulnerability at the threshold of 1 m/s in older cancer patients aged 65 and older for two main reasons. These data were based on 45 prospective cohort studies and involved 46845 older ambulatory people with mean age ranging from 65 to 89.6 years. First, GS was significantly associated with adverse events of frailty such as disability, falls, hospitalizations and death. Second, GS is a single item and may be a quick, easy, and reliable alternative screening test [7].

Here, we hypothesized that GS could be used as a single tool to screen for vulnerability in older cancer patients.

We assessed the diagnostic performance of GS for assessing vulnerability in comparison to the G8 and G8 modified indices.

## RESULTS

### Patients

**Figure 1:**
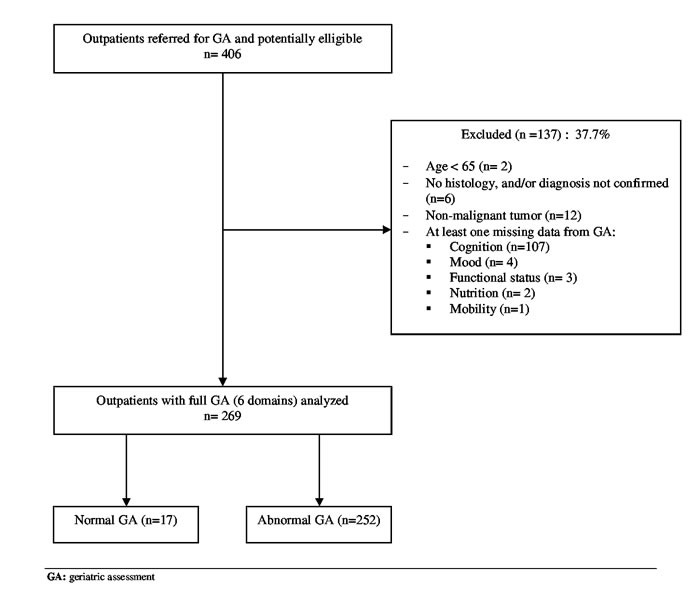
Flow the selection of patients

Among the 406 patients aged 65 and older with cancer between November 2013 and April 2016 who were referred to a geriatrician for a GA, 269 outpatients (66.3% 95%CI 61.4-70.8) were eligible for this study (Figure 1). We excluded 112/406 patients (27.6% 95%CI 23.3-32.2) because of missing data (at least one missing domain). Patients with and without missing data did not differ in mean age (81.3 years [5.9] *vs* 81.3 years [5.8], *P* = 0.97), mean gait speed (0.67 m/s [0.38] *vs* 0.71 m/s [0.37], *P* = 0.26), metastatic status (33.9% *vs* 39.4%, *P* = 0.31) or functional status assessed by the ECOG-PS (ECOG-PS > 1, 51.8% *vs* 40.9%, *P* = 0.05).

### Characteristics of patients with abnormal GA

The frequency of abnormal GA was 93.7% (n = 252/269) (95% CI 90.1-96.3). Overall, 94.4% had a solid cancer, and the two most common tumor sites were breast and colorectal (baseline characteristics in Table 1). Many patients had ECOG-PS ≤ 1 (56.7%). Dependency in IADL was the most common impaired domain. The mean (SD) GS was 0.69 (0.37 [IQR 0-1.53]), the median (IQR) G8 index was 11 [2.5-16], and median G8 modified index was 17 [0–35]. The median number of impaired domains was 3 [1–6]. The mean GS decreased significantly with increasing number of impaired domains (Table 2).

**Table 1:** Baseline clinical characteristics of 269 older cancer outpatients with geriatric assessment (GA)

Variables	All patients n=269	Patients with normal GA n=17 (6.3%)	Patients with abnormal GA n=252 (93.7%)	*P* value*
Age (y), mean (SD) [min-max]	81.3 (5.8) [65-97]	81.4 (5.4) [70-90]	81.3 (5.9) [65-97]	0.90
Gender (women)	148 (55)	8 (47.1)	140 (55.6)	0.49
Cancer site: Breast Colorectal Lung Liver Pancreas and bile duct Hematological malignancies Gynecological Urologic malignancies Esophagus and stomach Unknown primary site Others^*^	57 (21.2) 45 (16.7) 41 (15.2) 32 (11.9) 20 (7.4) 15 (5.5) 14 (5.2) 13 (5.0) 12 (4.4) 5 (2.0) 15 (5.5)	4 (23.5) 8 (47.0) 2 (11.8) 1 (5.9) 0 1 (5.9) 0 0 0 0 1 (5.9)	53 (21.0) 37 (14.7) 39 (15.5) 31 (12.3) 20 (8.0) 14 (5.5) 14 (5.5) 13 (5.2) 12 (4.8) 5 (2.0) 14 (5.5)	0.73
Metastatic:	106 (39.4)	9 (52.9)	97 (38.5)	0.30
ECOG-PS (>1):	110 (40.8)	1 (5.9)	109 (43.3)	0.001
G8 index (0-17): Median [IQR] ≤ 14	11 [2.5-17] 238 (88.4)	13 [10.5-17] 11 (64.7)	11 [2.5-16] 227 (90.1)	0.001
G8 modified index (0-35): Median [IQR] ≥ 6	17 [0-35] 231 (85.8)	3 [0-17] 6 (35.3)	17 [0-35] 225 (89.3)	< 0.0001
Gait speed (m/s): Mean (SD) [IQR] < 1 m/s	0.71 (0.37) [0-1.53] 206 (76.5)	1.07 (0.18) [0.79-1.33] 6 (35.3)	0.69 (0.37) [0-1.53] 200 (79.4)	< 0.0001 < 0.0001
Comorbidities (CIRS-G): Total > 14 No. of grade 3 (severe) ≥ 1 No. of grade 4 (very severe) ≥ 1	111 (41.2) 159 (59.1) 60 (22.3)	0 0 0	111 (44) 159 (63.1) 60 (23.8)	0.0001 < 0.0001 0.01
Dependency: IADL < 4/4 ADL ≤ 5/6	166 (61.7) 86 (31.9)	0 0	166 (65.9) 86 (34.1)	< 0.0001 0.001
Malnutrition: BMI < 21 kg/m^2^ Albumin level < 35 g/l (n=237)	34 (12.6) 73 (30.8)	0 0	34 (13.5) 73 (33)	0.14 0.003
Mobility: SPPB < 9/12	124 (46.1)	0	124 (49.2)	< 0.0001
Depressed mood: Mini-GDS ≥ 1	124 (46.1)	0	124 (49.2)	< 0.0001
Cognition: MMSE < 24/30	148 (55)	0	148 (58.7)	< 0.0001
No. of domains impaired: 0 1 2 3 4 5 6 median [IQR]	17 (6.3) 41 (15.2) 32 (11.9) 55 (20.4) 52 (19.3) 59 (21.9) 13 (4.8)	17 (100) 0 0 0 0 0 0	0 41 (16.3) 32 (12.7) 55 (21.8) 52 (20.6) 59 (23.4) 13 (5.2) 3 [1-6]	< 0.0001

Abbreviations: ADL: activity of daily living; BMI: body mass index; CIRSG: cumulative illness rating scale geriatric; ECOG-PS: eastern cooperative oncology group performans status; IADL: instrumental activity of daily living; IQR: interquartile range; mini-GDS: mini geriatric depression scale; MMSE: mini mental state examination; SPPB: short physical performance battery

* Chi-square test or Fisher's exact test for qualitative variables and Student t test or Wilcoxon's test for quantitative variables

** other: skin (melanoma/squamous carcinoma: 4), mesothelioma (3), sarcoma (3), anal (2), thymus (1), head and neck (1), duodenum (1)

**Table 2:** Gait speed (GS) by number of domains impaired in 269 older cancer outpatients

No. of impaired domains	No. of impaired domains (median [interquartile range])	Patients (n, %)	GS (m/s) (mean [SD])	*P* value*
- 0	3 [0-6]	- 17 (6.3)	- 1.07 (0.18)	< 0.0001
1		41 (15.2)	1.06 (0.22)	
2		32 (11.9)	0.96 (0.18)	
3		55 (20.4)	0.74 (0.30)	
4		52 (19.3)	0.62 (0.29)	
5		59 (21.9)	0.40 (0.31)	
6		13 (4.8)	0.22 (0.30)	

* Kruskal–Wallis test

### Correlation among GA tests (Table 3)

The number of impaired GA domains was inversely correlated with GS and G8 index — the Spearman rho for GS was -0.71 and the G8 index was -0.56 (both *P* < 0.0001) — but was positively and significantly correlated with the G8 modified index (Spearman rho = 0.55, *P* < 0.0001).

**Table 3:** Correlations among GS, G8 index and G8 modified index and GA domains

	Comorbidities	Dependency		Nutrition		Mobility	Mood	Cognition
	CIRSG total	IADL	ADL	BMI	Albumin level	SPPB / SPPB-modified	Mini-GDS	MMSE
GS	-0.28*	0.58*	0.59*	-0.08	0.34*	0.87* / 0.77*	-0.31*	0.41*
G8 index	-0.18*	0.53*	0.42*	0.37*	0.35*	0.44* / 0.40*	-0.34*	0.31*
G8 modified	0.24*	-0.56*	-0.43*	-0.14*	-0.35*	-0.42* / -0.38*	0.32*	-0.31*

Correlation: Spearman's rho test

* *P* < 0.05

### Diagnostic performance of the three tests to screen for abnormal GA

On varying the threshold of GS, the threshold of 1 m/s conferred the best sensitivity, with acceptable specificity (Table 4). The most sensitive tool was the G8 index but it had the lowest specificity. Specificities for GS and the G8 modified index were better than for the G8 index and were both similar. The NPV for the three tests was low. The negative likelihood ratio (NLR) for the G8 modified index was very good and acceptable for the G8 index and GS. With two GA domains impaired as the reference, GS sensitivity increased substantially and specificity remained acceptable; the specificity of the G8 and G8 modified indices decreased substantially. The diagnostic performance of GS was unchanged when it was excluded from the GA reference test and when incomplete cases were included (at least one domain missing). The sensitivity analysis by tumor site and metastatic status remained acceptable at the threshold of 1 m/s.

**Table 4:** Diagnostic performances of GS, G8 index and G8 modified index to screen for abnormal GA

Screening tools	Se (%) 95%CI	Sp (%) 95%CI	PPV (%) 95%CI	NPV (%) 95%CI	PLR 95%CI	NLR 95%CI
**At least 1 domain impaired in complete cases (n=252):**						
GS < 1.1 m/s	88.9 (84.3-92.5)	47.1 (23.0-72.2)	96.1 (92.8-98.2)	22.2 (10.1-39.2)	1.67 (1.07-2.63)	0.23 (0.12-0.43)
GS < 1 m/s	79.4 (73.8-84.2)	64.7 (38.3-85.8)	97.1 (93.8-98.9)	17.5 (9.1-29.1)	2.24 (1.17-4.29)	0.31 (0.20-0.48)
GS < 0.9 m/s	70.2 (64.2-75.8)	76.5 (50.1-93.2)	97.8 (94.4-99.4)	14.8 (8.1-23.9)	2.98 (1.26-7.05)	0.38 (0.28-0.53)
GS < 0.8 m/s	53.6 (47.2-59.9)	94.1 (71.3-99.9)	99.3 (96.0-100)	12.0 (7.0-18.8)	9.10 (1.35-61.18)	0.49 (0.41-0.58)
G8 index ≤ 14/17	90.1 (85.7-93.5)	35.3 (14.2-61.7)	95.4 (91.9-97.7)	19.4 (7.5-37.5)	1.39 (0.97-1.98)	0.28 (0.13-0.59)
G8 modified index ≥ 6/35	89.3 (84.8-92.8)	64.7 (38.3-85.8)	97.4 (94.4-99.0)	28.9 (15.4-45.9)	2.53 (1.32-4.82)	0.16 (0.10-0.27)
**At least 2 domains impaired in complete cases (n=211):**						
GS < 1.1 m/s	93.8 (89.7-96.5)	39.7 (27.0-53.4)	85.0 (79.7-89.3)	63.9 (46.2-79.2)	1.55 (1.25-1.92)	0.15 (0.08-0.28)
GS < 1 m/s	86.7 (81.4-91.0)	60.3 (46.6-73.0)	88.8 (83.7-92.8)	55.6 (42.5-68.1)	2.18 (1.58-3.01)	0.22 (0.14-0.32)
GS < 0.9 m/s	79.6 (73.5-84.8)	77.6 (64.7-87.5)	92.8 (88.0-96.1)	51.1 (40.2-61.9)	3.55 (2.19-5.76)	0.26 (0.19-0.35)
GS < 0.8 m/s	62.1 (55.2-68.7)	91.4 (81.0-97.1)	96.3 (91.6-98.8)	39.8 (31.5-48.7)	7.20 (3.09-16.75)	0.41 (0.34-0.50)
G8 index ≤ 14/17	93.4 (89.1-96.3)	29.3 (18.1-42.7)	82.8 (77.4-87.3)	54.8 (36.0-72.7)	1.32 (1.11-1.56)	0.22 (0.11-0.43)
G8 modified index ≥ 6/35	91.9 (87.4-95.2)	36.2 (24.0-49.9)	84.0 (78.6-88.5)	55.3 (38.3-71.4)	1.44 (1.18-1.75)	0.22 (0.12-0.39)
G8 index modified ≥ 7/35	89.1 (84.1-93.0)	41.4 (28.6-55.1)	84.7 (79.3-89.2)	51.1 (36.1-65.9)	1.52 (1.21-1.89)	0.26 (0.16-0.43)
G8 index modified ≥ 8/35	86.3 (80.9-90.6)	48.3 (35.0-61.8)	85.8 (80.4-90.2)	49.1 (35.6-62.7)	1.66 (1.29-2.15)	0.28 (0.18-0.43)
G8 index modified ≥ 9/35	81.0 (75.1-86.1)	48.3 (35.0-61.8)	85.1 (79.4-89.7)	41.2 (29.4-53.8)	1.56 (1.21-2.02)	0.39 (0.26-0.57)
G8 index modified ≥ 10/35	77.3 (71.0-82.7)	48.3 (35.0-61.8)	84.5 (78.6-89.3)	36.8 (26.1-48.7)	1.49 (1.15-1.93)	0.47 (0.32-0.67)
**At least 1 domain impaired and excluding GS (n=253):**						
GS < 1.1 m/s	88.9 (84.4-92.5)	50.0 (24.7-75.3)	96.6 (93.3-98.5)	22.2 (10.1-39.2)	1.77 (1.08-2.90)	0.22 (0.12-0.40)
GS < 1 m/s	79.4 (73.9-84.3)	68.8 (41.3-89.0)	97.6 (94.4-99.2)	17.5 (9.1-29.1)	2.54 (1.22-5.27)	0.29 (0.19-0.45)
GS < 0.9 m/s	70.0 (63.9-75.5)	75.0 (47.6-92.7)	97.8 (94.4-99.4)	13.6 (7.2-22.6)	2.79 (1.19-6.56)	0.40 (0.28-0.56)
GS < 0.8 m/s	53.4 (47.0-59.6)	93.8 (69.8-99.8)	99.3 (96.0-100)	11.3 (6.5-17.9)	8.53 (1.27-57.15)	0.49 (0.41-0.59)
G8 index ≤ 14/17	90.1 (85.8-93.5)	37.5 (15.2-64.6)	95.8 (92.4-98.0)	19.4 (7.5-37.5)	1.44 (0.98-2.11)	0.26 (0.12-0.54)
G8 modified index ≥ 6/35	89.3 (84.9-92.8)	68.8 (41.3-89.0)	97.8 (95.0-99.3)	28.9 (15.4-45.9)	2.85 (1.38-5.92)	0.15 (0.09-0.25
**At least 1 domain impaired and including complete and incomplete cases (n=364):**						
GS < 1.1 m/s	89.3 (85.6-92.2)	47.1 (23.0-72.2)	97.3 (94.9-98.8)	17.0 (7.6-30.8)	1.68 (1.07-2.64)	0.22 (0.12-41.0)
GS < 1 m/s	79.6 (75.1-83.6)	64.7 (38.3-85.8)	98.0 (95.6-99.3)	12.9 (6.6-22.0)	2.25 (1.18-4.30)	0.31 (0.21-0.47)
GS < 0.9 m/s	69.1 (64.1-73.9)	76.5 (50.1-93.2)	98.4 (96.0-99.6)	10.4 (5.7-17.1)	2.93 (1.24-6.94)	0.40 (0.29-0.54)
GS < 0.8 m/s	55.1 (49.8-60.3)	94.1 (71.3-99.9)	99.5 (97.3-100)	8.9 (5.2-14.1)	9.36 (1.39-62.85)	0.47 (0.40-0.56)
G8 index ≤ 14/17	90.1 (86.6-93.0)	35.3 (14.2-61.7)	96.8 (94.3-98.4)	14.3 (5.4-28.5)	1.39 (0.97-1.98)	0.28 (0.13-0.57)
G8 modified index ≥ 6/35	88.5 (84.7-91.6)	64.7 (38.3-85.8)	98.2 (96.1-99.3)	20.8 (10.8-34.1)	2.50 (1.31-4.77)	0.17 (0.11-28.0)
**At least 1 domain impaired in complete cases of breast cancer (n=53):**						
GS < 1.1 m/s	84.9 (72.4-93.3)	50.0 (6.8-93.2)	95.7 (85.5-99.5)	20.0 (2.5-55.6)	1.69 (0.63-4.55)	0.30(0.09-0.97)
GS < 1 m/s	71.7 (57.7-83.2)	75.0 (19.4-99.4)	97.4 (86.5-99.9)	16.7 (3.6-41.4)	2.86 (0.52-15.8)	0.37 (0.18-0.76)
GS < 0.9 m/s	67.9 (53.7-80.1)	75.0 (19.4-99.4)	97.3 (85.8-99.9)	15.0 (3.2-37.9)	2.71 (0.49-14.9)	0.42 (0.21-0.85)
GS < 0.8 m/s	58.5 (44.1-71.9)	100 (83.8-100)	100 (83.8-100)	15.4 (4.4-34.9)	-	0.41 (0.30-0.57)
**At least 1 domain impaired in complete cases of colorectal cancer (n=37):**						
GS < 1.1 m/s	83.8 (68.0-93.8)	62.5 (24.5-91.5)	91.2 (76.3-98.1)	45.5 (16.7-76.6)	2.23 (0.90-5.52)	0.25 (0.10-0.64)
GS < 1 m/s	73.0 (55.9-86.2)	62.5 (24.5-91.5)	90.0 (73.5-97.9)	33.3 (11.8-61.6)	1.94 (0.77-4.86)	0.43 (0.20-0.91)
GS < 0.9 m/s	62.2 (44.8-77.5)	75.0 (34.9-96.8)	92.0 (74.0-99.0)	30.0 (11.9-54.3)	2.48 (0.72-8.47)	0.50 (0.28-0.89)
GS < 0.8 m/s	48.6 (31.9-65.6)	87.5 (47.3-99.7)	94.7 (74.0-99.9)	26.9 (11.6-47.8)	3.89 (0.60-25.0)	0.58 (0.39-0.88)
**At least 1 domain impaired in complete cases of lung cancer (n=39):**						
GS < 1.1 m/s	87.2 (72.6-95.7)	50.0 (1.3-98.7)	97.1 (85.1-99.9)	16.7 (0.4-64.1)	1.74 (0.43-7.0)	0.25 (0.05-1.28)
GS < 1 m/s	74.4 (57.9-87.0)	50.0 (1.3-98.7)	96.7 (82.8-99.9)	9.1 (0.2-41.3)	1.48 (0.36-6.01)	0.51 (0.11-2.26)
GS < 0.9 m/s	64.1 (47.2-78.8)	50.0 (1.3-98.7)	96.2 (80.4-99.9)	6.7 (0.2-31.9)	1.28 (0.31-5.22)	0.71 (0.16-3.05)
GS < 0.8 m/s	46.2 (30.1-62.8)	100 (9.4-100)	100 (74.0-100)	8.7 (1.1-28.0)	-	0.53 (0.40-0.72)
**At least 1 domain impaired in complete cases of localized status (n=155)**						
GS < 1.1 m/s	87.7 (81.5-92.5)	37.5 (8.5-75.5)	96.5 (91.9-98.8)	13.6 (2.9-34.9)	1.40 (0.81-2.40)	0.32 (0.12-0.87)
GS < 1 m/s	77.4 (70.0-83.7)	62.5 (24.5-91.5)	97.6 (93.0-99.5)	12.5 (4.2-26.8)	2.06 (0.84-5.07)	0.36 (0.19-0.66)
GS < 0.9 m/s	67.1 (59.1-74.4)	75.0 (34.9-96.8)	98.1 (93.4-99.8)	10.5 (4.0-21.5)	2.68 (0.80-8.95)	0.43 (0.27-0.69)
GS < 0.8 m/s	51.6 (43.5-59.7)	100 (51.8-100)	100 (93.3-100)	9.6 (4.3-18.1)	-	0.48 (0.41-0.56)
**At least 1 domain impaired in complete cases of metastatic status (n=97):**						
GS < 1.1 m/s	90.7 (83.1-95.7)	55.6 (21.2-86.3)	95.7 (89.2-98.8)	35.7 (12.8-64.9)	2.04 (0.98-4.24)	0.16 (0.07-0.39)
GS < 1 m/s	82.5 (73.4-89.4)	66.7 (29.9-92.5)	96.4 (89.8-99.2)	26.1 (10.2-48.4)	2.47 (0.97-6.26)	0.26 (0.14-0.49)
GS < 0.9 m/s	75.3 (65.5-83.5)	77.8 (40.0-97.2)	97.3 (90.7-97.2)	97.3 (90.7-99.7)	3.38 (0.99-11.5)	0.31 (0.19-0.52)
GS < 0.8 m/s	56.7 (46.3-66.7)	88.9 (51.8-99.7)	98.2 (90.4-100)	16.0 (7.2-29.1)	5.10 (0.79-32.6)	0.48 (0.35-0.67)

Abbreviations: Se: sensitivity; Sp: specificity; PPV: positive predictive value; NPV: negative predictive value; PLR: positive likelihood ratio; NLR: negative likelihood ratio.

**Figure 2:**
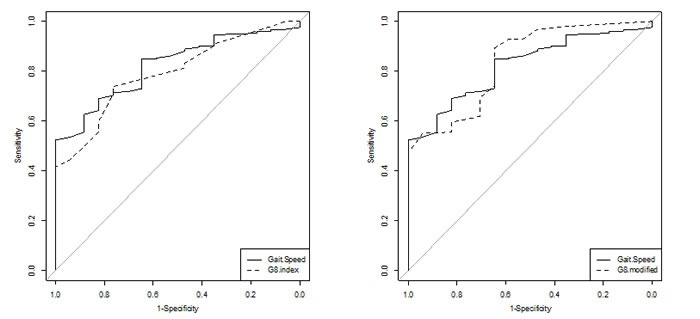
Receiver operating characteristic curves to screen for abnormal GA in 252 older cancer outpatients Comparison of GS with G8 index and G8 modified index.

Figure 2, compares the discriminative value assessed by AUC the for the three tools for one GA domain impaired. The AUC for the three tools was 82% (95% CI 74.0-90.0) for GS, 79% (70.0-88.0) for the G8 index and 84% (74.0-92.0) for the G8 modified index. The three tools had very good discrimination; the best in absolute value was the GS and G8 modified indices. The AUCs for the three tests did not differ in screening abnormal GA (GS/G8, *P* = 0.50; GS/G8 modified, *P* = 0.79)

## DISCUSSION

This is the first study to assess the diagnostic performance of GS as a screening tool in geriatric oncology and the first external validation of the G8 modified index. The GS and G8 modified indices had good diagnostic performance as tools to screen abnormal GA in a population of older outpatients with untreated cancer and with several tumor locations. Indeed, in the validation against the reference test (at least 1 abnormal test result), sensitivity was good, 79.4% and 89.3% for GS and the G8 modified index. The specificity was moderate, 64.7%, for both tools. In contrast, the sensitivity of the G8 index was very good, 90.1%, but the specificity was very low, 35.3%. The threshold of 1 m/s for GS maximized sensitivity with moderate specificity, and the proportion of patients with impaired GS was 76.5%.

This result agrees with the available data for community-dwelling older people, showing 1 m/s GS strongly associated with complications related to frailty (early death, falls, disability and hospitalization/institutionalization) [6] and predicting 10-year survival in a large meta-analysis of studies involving 34,485 community-dwelling older people aged 65 years and older [8]. Therefore, GS may be an indicator of vitality because of its multidimensional integration of disturbances in multiple organ systems [8].

In our study, the frequency of vulnerability (at least one abnormal GA domain) was very high (93.7%). The frequency of vulnerability varied widely across studies conducted in geriatric oncology probably because of the heterogeneity of older cancer patients included in these studies (various cancers or not, solid cancers or haematological malignancies, threshold of age at inclusion), variation in definition of abnormal GA (1 or 2 domains impaired), number of geriatric domains assessed (3-8), and scales used to assess each geriatric domain and the threshold used for each scales [2]. A further analysis of these previous studies showed that when mobility was assessed in a full GA by using walking tests (timed get-up-and-go test or short physical performance battery), the frequency of vulnerability was higher and varied from 60% to 94% [9–15]. In contrast, when mobility was not assessed, the frequency of vulnerability was substantially lower, from 28% to 88% [16–23]. However, in our study, when mobility was excluded from the definition of abnormal GA, the proportion of vulnerable patients was unchanged. In addition, in our study, the high frequency of vulnerability was probably due to a residual selection bias of patients. Indeed, patients were referred for GA because they were suspected to be frail, which increased the proportion of vulnerable patients and limited that of non-vulnerable patients. Therefore, the NPV for the three tests was low.

As in the study by Soubeyran et al., we found a similar and good sensitivity, as well as AUC, for the G8 index, but the specificity was very low [9, 10]. Although we used the same definition of vulnerability and same number of domains of GA as the previous study, we included only outpatients and had a greater proportion of metastatic cancers, and the scales used substantially differed between the studies. Indeed, in our study, nutrition was assessed by albumin level and BMI, whereas in the Soubeyran et al. study, nutrition was assessed by using the Mini-Nutritional Assessment. However, low BMI was uncommon in our study (12.6%). Moreover, the assessment of mobility and mood differed, but the two studies found similar correlations between the different screening tools. These differences may explain the low specificity of the G8 index in our study. For the same reasons, we found a lower specificity for the G8 modified index.

Furthermore, GS seems to have similar diagnostic performance as the Vulnerable Elders Survey 13 (VES-13), another screening tool for vulnerability widely used in geriatric oncology studies with sensitivity from 57% to 87% and specificity from 62% to 100% [2], but not used in our study.

GS was robust to sensitivity analyses involving changes in the definition of abnormal GA, missing data handling, and variation of threshold. Moreover, it showed homogeneity across tumor sites. The joint analysis of the performance of the three tools showed similar and good diagnostic performance, with the best specificity observed for GS and the G8 modified index. Although the proportion of people with abnormal GA may appear high in our study, it should be noted that patients were referred for a GA because they were suspected to be frail and that more than one third of patients was metastatic. We expect in a general population of older cancer patients a smaller proportion of frail and metastatic patients leading to lower estimates of at risk patients. The three tools being comparable for diagnostic performance, what benefits would it have to use gait speed? First, gait speed may serve as a single-item screening tool to determine which patients need a geriatric multidisciplinary approach to care [24]. Indeed, gait speed may serve as a marker of physiological reserve and potentially could quantify overall health status [25]. Second, gait speed might be used to identify older cancer adults with increased risk of early mortality [26]. Third, gait speed might be monitored over time, with a decline indicating an adverse event that requires evaluation and follow-up. However inadequate space and the need of chronometer could be obstacles to the routine assessment in oncologic setting. Furthermore, our study is the first external validation of the G8 modified index, which showed very good diagnostic performance (discrimination ability: 84%) to detect vulnerable patients, with similar sensitivity (89.3%).

However, our study has several limitations. First, liver cancer was strongly represented. Indeed, one of the recruitment centers (Jean Verdier hospital) specializes in hepatocellular carcinoma. Also, we did not include inpatients because of difficulties in measuring GS with inpatients. We recognize a selection bias of patients in that included patients were referred for GA. Finally, the relatively small number of patients per tumor site led to wide confidence intervals of performance indices in subgroup analyses.

In practice, GS and the G8 modified index could be used in routine practice to screen for vulnerability in older cancer outpatients and to identify patients who need a GA. Further studies are needed to validate the performance of GS in an external cohort and measure the acceptability of these tools by cancer physicians in routine practice.

Conclusions: GS < 1 m/s with a single measure could be a new tool to screen vulnerability in older cancer outpatients for selecting patients for a GA. The external validation of the G8 modified index was very good. Indeed, GS and the G8 modified index are as good as the G8 index in detecting vulnerability, but external validation of GS is needed to confirm these data and to measure the acceptability of this tool in routine practice.

## MATERIALS AND METHODS

We followed the Standards for Reporting Diagnostic Accuracy studies (STARD) recommendations [27].

### Study design and population

The Physical Frailty in Elderly Cancer patients (PF-EC) survey is a prospective observational bicentric cohort study that started in November 2013. All consecutive older outpatients referred for geriatric oncology assessment in two university hospitals in the greater Paris area, Avicenne hospital (Bobigny, France) and Jean Verdier hospital (Bondy, France), were included. Patients were referred by oncologists, radiotherapists, surgeons, or other specialists when a diagnosis of cancer was highly suspected or confirmed histologically, and when a frailty was suspected before a therapeutic decision.

We included every outpatient age 65 and older with cancer confirmed histologically (except for some hepatocellular carcinomas, the diagnosis of which can be made by dynamic imagery in patients with underlying cirrhosis [28]), regardless of cancer type, stage or treatment, who presented up to April 2016. Patients with at least one domain of geriatric assessment missing were excluded. The inclusion date was the date of the first geriatric oncology visit.

We performed a cross-sectional analysis of baseline data of the PF-EC cohort.

Informed consent was obtained from studied patients before inclusion.

The study was approved by a local ethics committee (CLEA, Avicenne Hospital, Bobigny, France).

### Tests methods

All tests methods were performed at the first geriatric oncology visit during the GA.

GS was measured for patients walking over a short distance (4 m) at a usual pace [6]. Patients had to walk along a corridor with the following directions: “Please begin walking at your normal pace” after the order “Go.” They walked for 2 m before the measurement began and stopped 2 m after the end of the measurement (8 m in total). Patients could use their cane if necessary. GS was measured by use of a chronometer by dividing the distance in meters (4 m) by the time in seconds (m/s). The best of two measures was retained. If a patient could not walk, the GS was scored as “0”. A slow GS was defined as < 1 m/s [6].

The G8 index is used to assess the risk of vulnerability in older cancer patients and includes eight items: appetite changes, weight loss, mobility, neuro-cognitive problems, body mass index (BMI), number of medications, self-reported health and age. The total G8 index score ranges from 0 to 17. The cutoff score for an “impaired” reference test G8 score is ≤ 14 [10].

The G8 modified index includes six items: weight loss, neuro-cognitive problems, number of medications, self-reported health, performance status and history of heart failure or coronary artery disease. The total G8 modified index score ranges from 0-35. The cutoff value for an “impaired” reference test G8 modified score is ≥ 6 [5].

### GA reference test (GA)

For each patient, a geriatrician and a nurse specialized in geriatric oncology (FP and AF) performed the GA at the first geriatric oncology visit, following the recently updated recommendations of the SIOG [29]. The assessment involved the following six domains. Comorbidities were assessed by the Cumulative Illness Rating Scale-Geriatric (CIRSG). A total score dichotomized by a median (> 14) or at least one comorbidity grade 3 (severe) or grade 4 (very severe) condition, excluding the current cancer, was considered impaired [30]. Dependency was defined by an Activities of Daily Living (ADL) score ≤ 5/6 and/or an Instrumental ADL (IADL) score simplified to four items (phone, transports, medications, and financial) < 4/4 [31, 32]. Malnutrition was defined by BMI < 21 kg/m^2^ and/or albumin level < 35 g/l [33]. Albumin level was measured by the immuno-turbidimetric assay method during the first 3 weeks after the GA. Mobility was assessed by the Short Physical Performance Battery (SPPB) score, a composite walking test that includes GS measurement (0-4 points), balance (0-4 points) and rising from a chair (0-4 points). Mobility was considered impaired with SPPB score < 9/12 [34]. Mood impairment was defined by a Mini-Geriatric Depression Scale (Mini-GDS) score of at least 1/4 [35]. Cognition impairment was defined by a Mini Mental State Examination (MMSE) score < 24/30 [36]. Abnormal GA was defined as an impaired score on at least one of the six domains.

### Covariate data collected during the GA

Data collected during the GA included age and gender; cancer characteristics (site, tumor extension: locations or metastatic/diffuse); Eastern Cooperative Oncology Group performance status (ECOG-PS), classified as not impaired (0-1) or impaired (2-4); and number of impaired domains of the GA.

### Outcome

The primary outcome was the the vulnerability defined by an abnormal GA.

### Statistical analysis

Qualitative data are described with number (%) and quantitative data with mean (SD) (or median, interquartile range [IQR]). Comparisons involved chi-square test or Fisher's exact test for qualitative variables and Student *t* test or Wilcoxon's test for quantitative variables. We assessed correlation by the Spearman rho test. Correlation was considered very low (0-0.2), low (0.2-0.4), moderate (0.4-0.6), strong (0.6-0.8) or very strong (> 0.8). Diagnostic performance was assessed by sensitivity, specificity, positive predictive value (PPV), NPV, positive likelihood ratio (PLR) and negative likelihood ratio (NLR). The accuracy of the screening tools was analysed by the area under the receiver operating characteristic curve (AUC). Comparison of AUCs involved bootstrapping the difference in AUC two-by-two and testing it with a Z-test.

Sensitivity analyses were performed to test the robustness of the results by 1) varying thresholds of gait speed (0.8, 0.9, 1 and 1.1 m/s), 2) using a cutoff of ≥ 2 impaired tests to define abnormal GA (reference test), 3) excluding GS measurement from the reference GA to minimize incorporation bias (we used an adapted SPPB test [0–8] with a threshold of 6 to define mobility impairment), 4) including incomplete cases (i.e., patients with at least one GA domain missing [considering abnormal GA with at least one impaired score among available GA domains and normal GA with all normal scores among available GA domains]), and 5) by cancer site (breast, colorectal and lung) and metastatic status (localized, metastasis).

All tests were two-sided, with *P*<0.05 considered statistically significant. Data were analysed by using R v3.2.2 (R Foundation for Statistical Computing, Vienna, Austria, http://www.R-project.org)
